# Supplementary material for: Toxicity Reduction of Euphorbia kansui Stir-Fried with Vinegar Based on Conversion of 3-O-(2′E,4′Z-Decadi-enoyl)-20-O-acetylingenol
Source: Molecules. 2019 Oct 22;24(20):3806. doi: 10.3390/molecules24203806 (PMC6832248; doi:10.3390/molecules24203806)
Supplement: Supplementary file 1 [file molecules-24-03806-s001.pdf]

Supplementary Materials

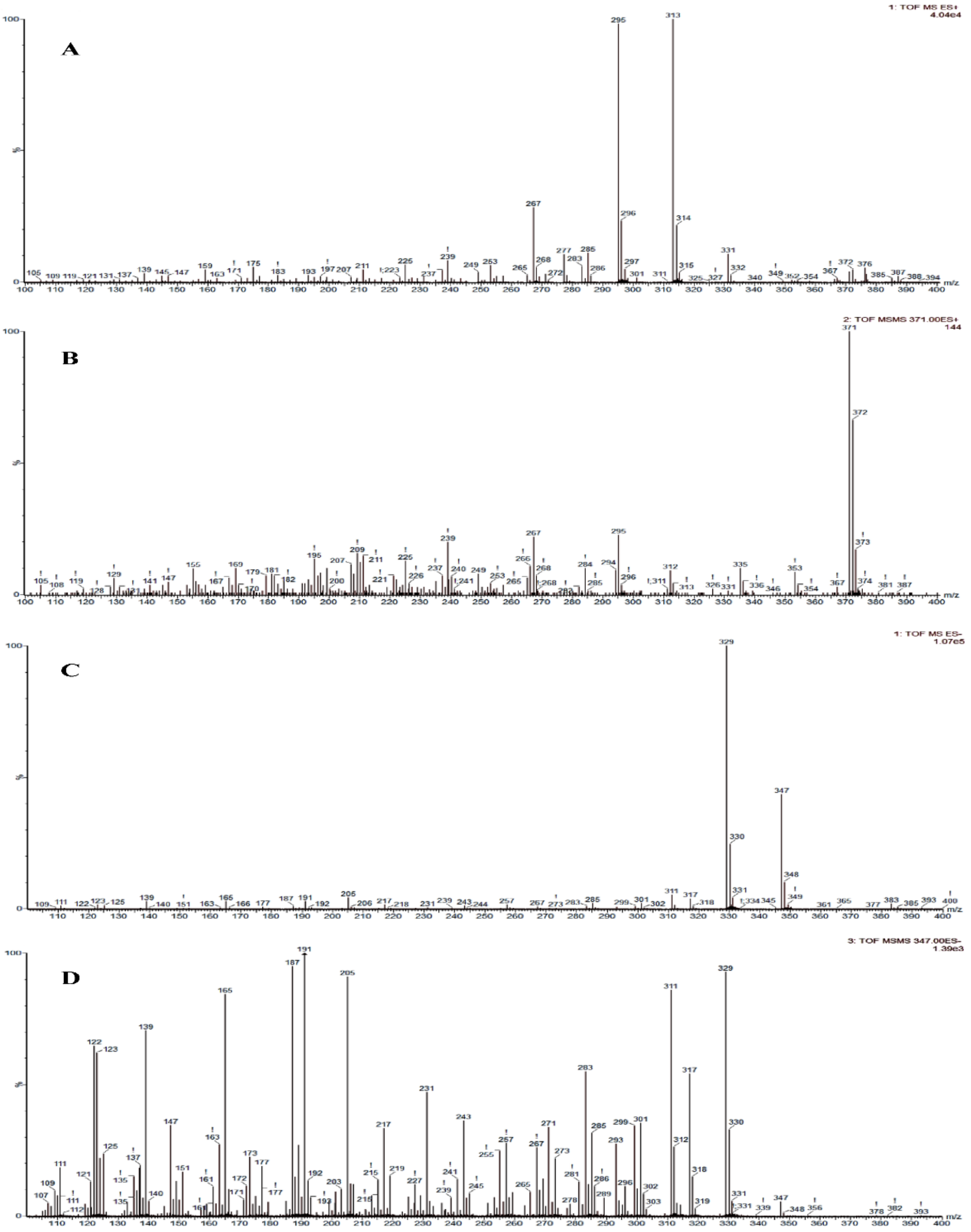

Figure. S1: Positive and negative ion mass spectrum of hydrolysate. A: 1 st ESI MS spectrum of positive; B: 2 nd ESI MS spectrum of positive; C: 1 st ESI MS spectrum of negative; D: 2 nd ESI MS spectrum of negative

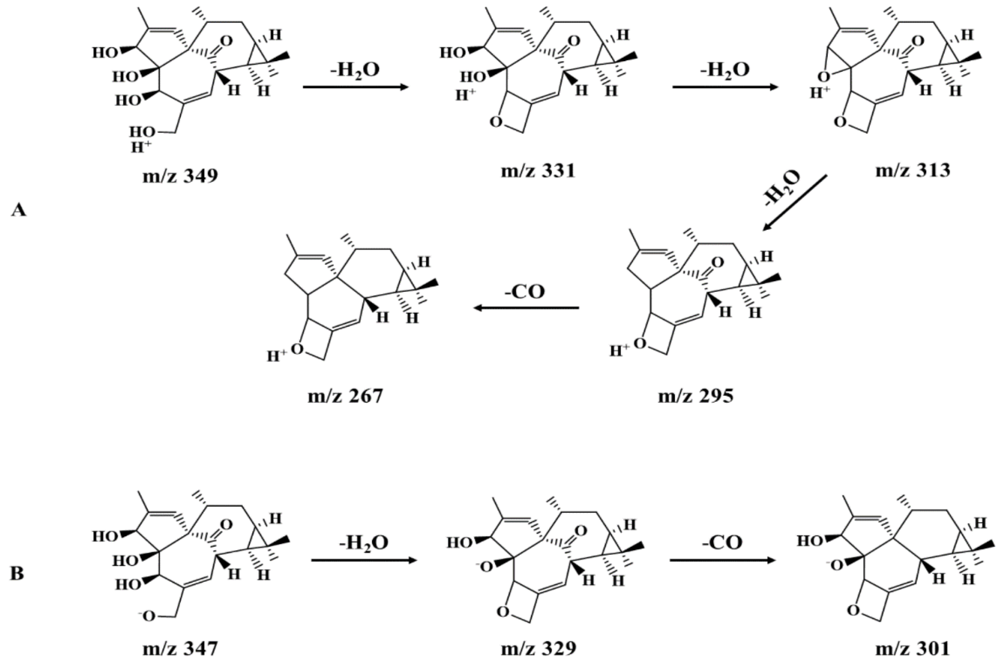

Figure. S2: Positive and negative ion mode mass spectrometry fragmentation process of hydrolysate. A: positive; B: negative

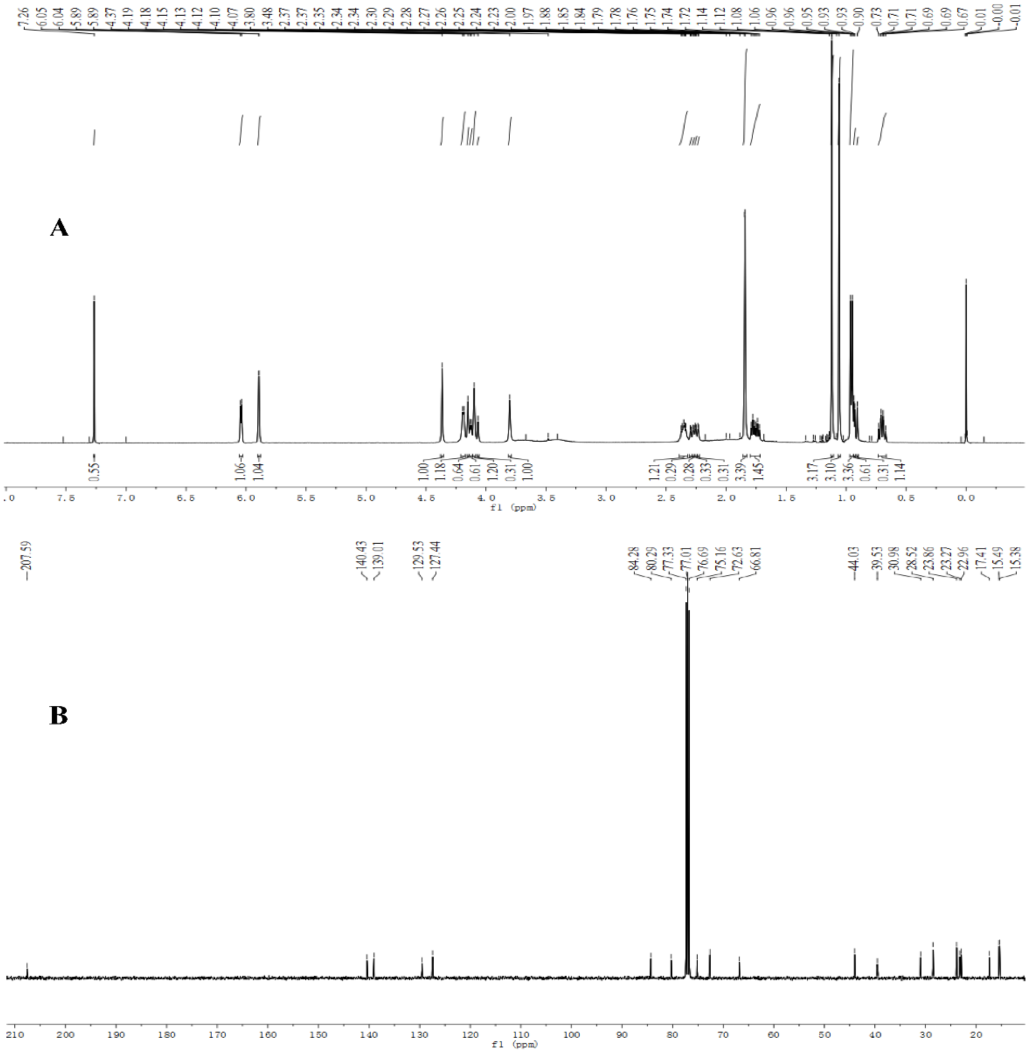

Figure. S3: The ^{1}H-NMR and ^{13}C-NMR of hydrolysate. A: ^{1}H-NMR; B: ^{13}C-NMR

Table S1. ^{1}H-NMR and ^{13}C-NMR data of ingenol

Position	ingenol	ingenol[19]	Position	ingenol	ingenol[18,19]
H-1	5.89 (d, *J*=1)	5.88 (d, *J*=1)	C-1	129.53	129.4
H-3	4.37 (s)	4.37 (s)	C-2	140.43	140.4
H-5	3.80 (brs)	3.80 (brs)	C-3	80.29	80.2
H-7	6.04 (d, *J*=5)	6.04 (d, *J*=5)	C-4	84.28	84.3
H-8	4.12 (m)	4.12 (m)	C-5	75.16	75.1
H-11	2.34 (m)	2.34 (m)	C-6	139.01	139.1
H-12	2.26 (ddd, *J*=15, 8, 3)	2.26 (ddd, *J*=15, 8, 3)	C-7	127.44	127.4
H-12'	1.76 (m)	1.76 (m)	C-8	44.03	44.0
H-13	0.71 (dd, *J*=15, 8)	0.70 (dd, *J*=15, 8)	C-9	207.59	207.7
H-14	0.93 (m)	0.93 (m)	C-10	72.63	72.7
H-16	1.06 (s)	1.06 (s)	C-11	39.53	39.5
H-17	1.12 (s)	1.12 (s)	C-12	30.98	31.0
H-18	0.95 (d, *J*=7)	0.95 (d, *J*=7)	C-13	23.27	23.3
H-19	1.84 (s)	1.84 (s)	C-14	22.96	23.0
H-20	4.18 (m)	4.18 (m)	C-15	23.86	23.8
H-20'	4.07 (m)	4.08 (m)	C-16	28.52	28.5
			C-17	15.38	15.4
			C-18	17.41	17.4
			C-19	15.49	15.5
			C-20	66.81	66.8

Record in $CDCl_3$, 400 MHz for ^{1}H, 100 MHz for ^{13}C, δ in ppm, J = Hz.

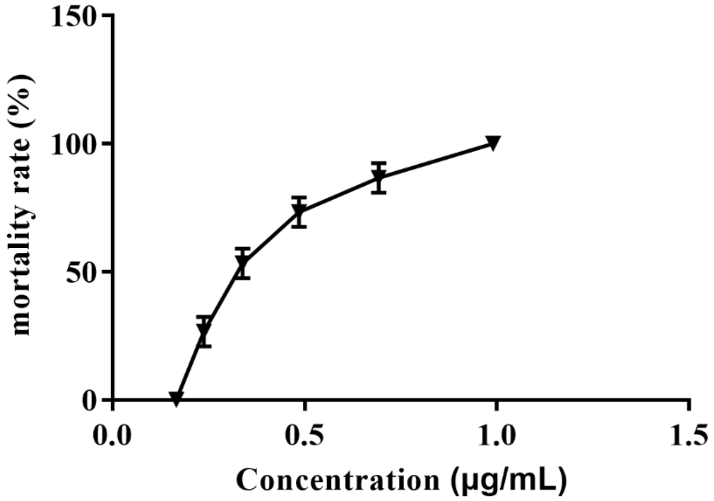

Figure. S4: The acute toxicity curves of 3-O-EZ in zebrafish embryos
